# The value of hsa_circ_0058514 in plasma extracellular vesicles for breast cancer

**DOI:** 10.3389/fonc.2022.995196

**Published:** 2022-11-01

**Authors:** Jiani Liu, Xinyu Peng, Yang Yang, Yao Zhang, Meng Han, Xiaohui Shi, Jie Zheng, Tong Li, Jinxia Chen, Weihua Lv, Yunjiang Liu, Yixin Qi, Lei Zhang, Qi Liu

**Affiliations:** ^1^ Department of Breast Surgery, The First Hospital of Qinhuangdao, Qinhuangdao, China; ^2^ Department of Gastrointestinal Surgery, Affiliated Hospital of Hebei University, Baoding, China; ^3^ Department of Oncology, Affiliated Hospital of Hebei University, Baoding, China; ^4^ Department of Plastic Surgery, Hangzhou Xiaoshan Yaoran Medical Cosmetology Clinic Co. Ltd, Hangzhou, China; ^5^ Department of Breast Surgery, The Fourth Hospital of Hebei Medical University, Shijiazhuang, China; ^6^ Graduate school of Chengde Medical University, Chengde, China; ^7^ Clinical Laboratory of the Fourth Hospital of Hebei Medical University, Shijiazhuang, China; ^8^ School of Nursing, Hebei Medical University, Shijiazhuang, China

**Keywords:** hsa_circ_0058514, EVS, biomarker, plasma, extracellular vesicles

## Abstract

The aim of this study was to investigate the diagnostic value of hsa_circ_0058514 in plasma extracellular vesicles (EVs) in BC patients and its predictive value for neoadjuvant chemotherapy. The expression of hsa_circ_0058514 in a large sample of BC plasma and healthy subjects’ plasma was detected by qPCR, and the ROC curve was drawn to verify its diagnostic value as a plasma tumor marker. Furthermore, the association between the expression of hsa_circ_0058514 and clinicopathological characteristics before and after treatment was detected in the plasma of 40 pairs of BC patients undergoing neoadjuvant therapy. The expression level of hsa_circ_0058514 in the plasma of BC patients was significantly higher than that of healthy subjects. The ROC curve showed that plasma hsa_circ_0058514 ROC in differentiating non-metastatic BC and healthy people had better diagnostic efficiency than conventional tumor markers CA153, CA125, and CEA. In patients with neoadjuvant therapy, the decrease in plasma hsa_circ_0058514 value before and after treatment correlated with pathological MP grade (*r* = 0.444, *p* = 0.004) and imaging tumor regression value (*r* = 0.43, *p* = 0.005) positive correlation. The detection of hsa_circ_0058514 in both extracellular vesicles of BC cell culture medium and human plasma was demonstrated. Hsa_circ_0058514 is detected in the plasma from BC cells secreted in the form of vesicles. Hsa_circ_0058514 can be used as an early plasma biological indicator for the diagnosis of BC in clinical applications, with a higher risk of recurrence and metastasis, and as a predictor of the effect of neoadjuvant therapy to guide the clinical use of neoadjuvant therapy.

## Introduction

According to the reported Global Cancer Statistics 2020 (GLOBOCAN) estimates of incidence and mortality worldwide by the International Agency for Research on Cancer, female BC has surpassed lung cancer as the most common cancer, and the estimated data of the morbidity and mortality showed that there were 2.3 million new cases annually, accounting for 11.7% of all new cancers (1). About 70%–80% of patients with early-stage non-metastatic BC are curable, but advanced BC with distant organ metastasis is considered to be incurable by current treatments, due to the poor prognosis, with a 26% 5-year survival rate (2). Based on the presence or absence of progesterone receptors, estrogen receptors, and human epidermal growth factor receptor-2 (HER-2), BC can be roughly divided into three different subtypes, namely, intraluminal (luminal A/B) type (positive hormone receptor), HER-2 type (overexpression of HER-2), and triple-negative BC (TNBC) (the loss of all three receptors) (3). Compared with luminal and HER2 subtypes, TNBC manifests more aggression, with a poorer prognosis and higher metastatic potential (3).

Because of the improvement in people’s health awareness and the advancement of diagnosis level, more and more BCs can be detected and treated early, but due to the systemic and complex nature of cancer, there are still many challenges in diagnosis. Traditional cancer detection methods, such as tissue biopsy, are not comprehensive enough to capture the entire genomic landscape of breast tumors. However, with the introduction of new technologies, the use of liquid biopsy has been more popular, resulting from the improvements in all aspects of BC management, including early screening and diagnosis, prognosis prediction, early detection of recurrence, and assessment of disease progression and response to treatment by continuous sampling and effective longitudinal monitoring (4). Compared with tissue biopsy, blood biopsy sampling as a liquid biopsy has the following advantages: low trauma, repeatability, easy operation, and dynamic monitoring during treatment (5). *Via* liquid biopsy sampling, various components of tumor cells released into the blood circulation can be analyzed, including circulating tumor cells (CTCs), circulating tumor DNA (ctDNA), cell-free RNA, tumor-induced platelets, and outer vesicles (5). However, nonspecific tumor markers can be used to achieve early diagnosis, treatment, and monitoring of treatment effects for improving the disease-free survival outcome of patients.

Circular RNA (circRNA) is a kind of non-coding RNA (ncRNA) that has been recently rediscovered. Different from linear RNA with a 5’ cap and a 3’ tail, circRNA is a single-stranded covalently closed circular transcript, only a few of which have been discovered in different organisms in the past 30 years (6). At present, many circRNAs are also found in human body fluids, such as plasma, urine, and saliva (6). Therefore, circRNAs have attracted interest as potential novel diagnostic and prognostic biomarkers for BC. At present, serum biomarkers used in the diagnosis and monitoring of BC include CEA, CAl53, and CA125, which have low sensitivity and specificity for the diagnosis of intermediate and early BC without recurrence and metastasis (7). There are no current data available that recommend the use of CA153, CEA, and CA125 to monitor treatment effectiveness.

Previous literature showed that circAGFG1, one of the circRNAs, is highly expressed in TNBC and associated with the poor prognosis outcome of TNBC, with a circBase ID of hsa_circ_0058514, located in chr2: 228356262-228389631 (8). This gene is highly expressed in cancer tissues and is a cancer-promoting gene (8). This study was the first to verify the expression of hsa_cir c_0058514 in BC plasma and to verify its clinical value and secretion mechanism as a tumor marker.

## Materials and methods

### The enrollment of subjects and the collection of plasma and tissue samples

Fasting venous blood was collected from BC patients admitted to the Fourth Hospital of Hebei Medical University from September 2019 to November 2020. Those who had received chemotherapy, radiotherapy, or targeted or interventional therapy in the past, and those with hypertension, diabetes, coronary heart disease, and other cancer histories were excluded. A total of 135 cases were excluded from our study. Intraoperative tumors and adjacent tumor tissues were collected from 38 cases without preoperative adjuvant therapy. Preoperative fasting venous blood were collected from 40 patients had after neoadjuvant treatment. Fasting venous blood was collected from healthy people who underwent a physical examination at the Fourth Hospital of Hebei Medical University from September 2020 to November 2020, and 95 cases were matched according to age. This study was approved by the Medical Ethics Committee of the Fourth Hospital of Hebei Medical University, and all subjects signed the informed consent.

### Miller Payne pathology evaluation system after neoadjuvant BC

Evaluation of residual invasive tumor cell abundance in primary breast foci after neoadjuvant therapy was performed by comparing hollow needle aspiration specimens with surgical specimens after treatment. MP grade 1: no change in invasive cancer cells or only individual cancer cells changed, and the overall number of cancer cells did not decrease. MP grade 2: invasive cancer cells were slightly reduced, but the total number was still high, and the reduction of cancer cells did not exceed 30%. MP grade 3: 30%–90% reduction in invasive cancer cells; MP grade 4: invasive cancer cells are significantly reduced by more than 90%, with only scattered small clusters of cancer cells or single cancer cells remaining. MP grade 5: the original tumor bed has not been infiltrated with cancer cells; there may be ductal carcinoma *in situ*.

### Quantitative real-time polymerase chain reaction

Plasma samples were isolated from 4 ml of peripheral blood using BD Vacutainer tubes (EDTA-K2 acted as anticoagulation) (BD, New Jersey, USA), and 4 ml of fasting venous blood was collected from healthy subjects and BC patients. Samples were centrifuged within 1 h (centrifugation condition: 4°C, 1,500 rpm for 10 min), and plasma was sucked with a 1-ml de-enzyme pipette tip, transported to the laboratory on ice, and centrifuged in low-temperature high-speed centrifuge within 6 h (centrifugation condition: 4°C, 15,000 rpm for 10 min). The supernatant was carefully aspirated with a de-enzyme pipette tip, aliquoted into 1.5-ml de-enzymatic EP tubes (AXYGEN, JIANGSU, China), 300 μl per tube, and stored in a −80°C refrigerator for later use. Postoperative plasma samples were collected about 2 weeks after curative resection for breast cancer. Plasma was collected preoperatively after neoadjuvant chemotherapy.

Total RNA in the plasma of BC patients and healthy volunteers was extracted by the TIANamp Virus RNA Kit (TIANGEN, Beijing) according to the instructions of the manufacturer. The purity and concentration of total RNA samples were detected by a NanoDrop 1000 instrument spectrophotometer (Thermo Scientific, Waltham, MA, USA). The average concentration of plasma-extracted RNA was 130–150 ng/μl; A260/280 was nearly 2.0. Total RNAs were reversely transcribed into cDNA using First-Strand cDNA Synthesis Super Mix (Beijing Quanshijin Biotechnology Co., Ltd.), and qRT-PCR was carried out using Top Green qPCR Super Mix (Beijing Quanshijin Biotechnology Co., Ltd.) and the Applied Biosystems Real-Time PCR System. The expression level of hsa_circ_0058514 in the plasma was determined by qRT-PCR assay using the sequences of the primers as listed in **Table S1**. Thermal cycling conditions were as follows: an initial 5-min step at 95°C followed by 45 cycles of 10 s denaturing at 95°C, 35 s annealing at 55°C, and 15 s extension at 95°C. The final step was conducted at 55°C for 1 min. Each sample was tested in triplicate for the last calculation and run with a non-template control (NTC) composed of sterile water instead of cDNA. The results of qRT-PCR analysis were presented using the 2^−ΔΔCT^ method, and the relative expression level of hsa_circ_0058514 was normalized to the GAPDH expression. To verify the qRT-PCR products of the plasma circRNAs, 5 μl of the product mixture was subjected to 2% agarose gel electrophoresis. The qRT-PCR product of each circRNA was sent for DNA sequencing at Sangon Biotech Co, Ltd. (Sangon, Shanghai, China).

### Enzyme-linked immunosorbent assay

The concentrations of serum tumor biomarkers (including CEA, CA125, and CA153) in BC patients and healthy controls were measured by the Cobas e602 system with the Elecsys CEA Assay kit (Roche Diagnostics, Basel, Switzerland) according to the manufacturer’s instructions. The cutoff values of CEA, CA153, and CA125 were 5 ng/ml, 37 U/ml, and 24 U/ml, respectively.

### Cell culture and exosome extraction

All the BC cells were obtained from the National Biomedical Laboratory Cell Resource Bank. MCF-10a cells were donated by the Department of Pharmacology, China Medical University. Before extracting exosomes from the cell culture medium, the medium was changed to a serum-free medium and cultured for 24 h, following the instructions of the TransExo Cell Media Exosome Kit (Transgen, Beijing). Human plasma exosome extraction followed the instructions of the Plasma Exosome Total RNA Extraction Kit (Transgen, Beijing). The exosome downstream isolated from 1 ml of cell supernatant was used for transmission electron microscopy and that from 6 ml of cell supernatant was used for Western blot and qPCR experiments. The extracted RNA was stored at −80°C for later use.

### Identification of extracellular vesicles of BC cells by transmission electron microscopy

The extracted EVs were resuspended in 30 μl of phosphate-buffered saline (PBS) and stored at 4°C. Excess liquid was removed from the edges of the beads with filter paper and stained with 1% uranyl acetate dye solution for 1 min. After drying at room temperature, EVs were observed with a transmission electron microscope (Hitachi ht7800).

### Western blotting

Protein was prepared with SDS-PAGE loading buffer. Equal amounts (30 μl) of protein samples were separated by a 12% gel using sodium dodecyl sulfate-polyacrylamide gel electrophoresis (SDS-PAGE) and transferred onto PVDF membranes (Millipore, Billerica, MA, USA). Monoclonal rabbit anti-CD9 (ET1601-9, Huaan Bio) and monoclonal rabbit anti-CD81 (ET1611-87, Huaan Bio) were incubated overnight at 4°C with the membranes. Immune complexes were detected by enhanced chemiluminescence (Cell Signaling Technology).

### Statistical analysis

Receiver operating characteristic (ROC) curves and the area under the ROC curve (AUC) were used to assess the diagnostic performance of hsa_circ_0058514. Differences in the values for plasma hsa_circ_0058514 between groups were determined by using the *t*-test and analysis of variance (ANOVA). Wilcoxon rank sum test and Pearson correlation analysis were used to determine correlations between expression levels of plasma hsa_circ_0058514 and clinical indexes. Statistical analysis was performed with SPSS (Version 22.0, IBM, USA) and presented graphically in GraphPad Prism 8.0. A *p*-value of 0.05 was statistically significant.

## Results

### Expression of plasma hsa_circ_0058514 in BC patients

**Figure 1:**
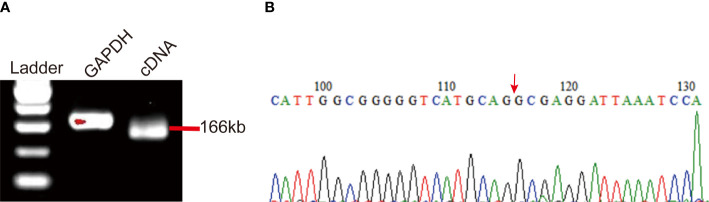
The identification and detection of hsa_circ_0058514 in BC patients’ plasma. **(A)** PCR product of hsa_circ_0058514 in agarose gel electrophoresis. **(B)** The structure of hsa_circ_0058514 searched in Circbase.

Hsa_circ_0058514 molecules showed an upregulated molecule in various cancer tissues, including triple-negative BC tissues. In the present study, in order to verify the expression of hsa_circ_0058514 in plasma samples of BC patients, a reverse primer spanning the circRNA cleavage point was used to amplify circRNA by qRT-PCR. The results of agarose gel electrophoresis showed that the fragment size of hsa_circ_0058514 was 166 kb (**Figure 1A**). The amplified product was sequenced, and it was further found that the hsa_circ_0058514 sequence had a reverse splicing structure, consistent with the structure in the circRNA database Circbase (**Figure 1B**). The specific detection of qRT-PCR indicated that the expression of hsa_circ_0058514 was in the peripheral blood of BC patients.

### The expression level of plasma hsa_circ_0058514 in BC patients and the association with clinicopathological features

**Figure 2:**
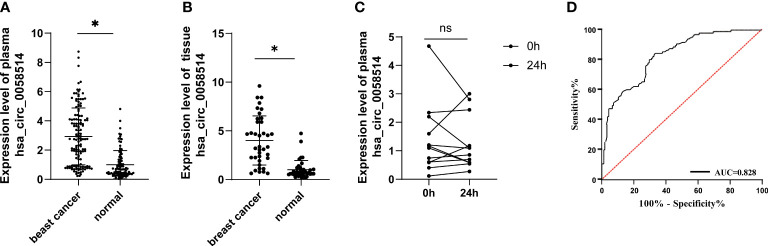
The expression level of hsa_circ_0058514 was higher in both plasma and cancer tissues of BC patients than healthy subjects. **(A)** The expression level of hsa_circ_0058514 detected in plasma and cancer tissues. **(B)** The stability experiment of hsa_circ_0058514 in plasma. **(C)** The stability experiment of hsa_circ_0058514. **(D)** The ROC curve of hsa_circ_0058514. *P < 0.05; ns, nonsignificant.

To further determine the expression level of hsa_circ_0058514, the comparison between plasma samples from 135 non-metastatic BC patients and 95 age- and sex-matched healthy volunteers showed a higher expression in patients, which was three times that of the control group (*p* < 0.001) (**Figure 2A**). On this basis, we analyzed the correlation between the expression of plasma hsa_circ_0058514 and the clinicopathological characteristics of BC patients. Results indicated that the higher expression was associated with lymph node metastasis (*p* < 0.001), high tumor stage (*p* < 0.05), high risk of BC recurrence after surgery (*p* < 0.001), and non-luminal molecular type (*p* < 0.05), but was not associated with age, tumor diameter, and high expression of Ki-67 correlation (**Table 1**). Meanwhile, we also detected the expression level of hsa_circ_0058514 in 38 pairs of BC samples and adjacent tissues. The expression level of hsa_circ_0058514 in BC tissues was consistent with the expression level in plasma. The expression level of normal breast tissue was four times that of normal breast tissue, and the difference was statistically significant (*p* < 0.001) (**Figure 2B**). The stability experiment of hsa_circ_0058514 showed that the fresh plasma of the same patient was placed at room temperature for 24 h to extract RNA, and the expression of hsa_circ_0058514 was not significantly different (*p* = 0.771), compared with 0 h directly (**Figure 2C**). At the optimal cutoff value of 0.509 with the value of sensitivity and specificity considered to be maximal for hsa_circ_0058514, the sensitivity and specificity were 84.70% and 66.70%, and the AUC value was 0.828 with a 95% CI of 0.776–0.880 (**Figure 2D**).

**Table 1:** The difference in hsa_circ_0058514 expression level associated with the clinical characteristics of BC patients.

	*N* (%)	△△CtM (IQR)	*z/χ*²	*p*
**Age (years)**				
** <50**	75 (55.6)	2.902 (3.414)	−1.275	0.202
** ≥50**	60 (44.4)	2.358 (3.217)		
**Tumor size (cm)**				
** ≤2**	69 (51.1)	2.154 (2.981)	−1.919	0.055
** >2**	66 (48.9)	3.111 (4.039)		
**Lymphatic metastasis**				
** With**	68 (50.4)	1.915 (1.943)	−4.791	<0.001
** Without**	67 (49.6)	3.777 (2.378)		
**Stage**				
** 0–1**	51 (37.8)	1.969 (1.921)	−3.443	<0.05
** 2–4**	84 (62.2)	3.452 (3.628)		
**Ki-67 expression**				
** Lower**	59 (43.7)	2.671 (3.185)	−1.251	0.211
** Higher**	76 (56.3)	2.843 (3.735)		
**Risk of BC recurrence after surgery**				
** Low**	23 (17.0)	1.786 (2.124)*	37.017	<0.001
** Medium**	72 (53.3)	2.052 (2.795)*		
** High**	40 (29.7)	4.590 (2.075)		
**Subtype**				
** Luminal type**	79 (58.5)	2.066 (2.934)*	13.426	<0.05
** HER-2 positive**	37 (27.4)	2.765 (2.660)		
** Triple negative**	19 (14.1)	4.949 (1.726)		

*, P < 0.05.

### The superior diagnostic value of plasma hsa_circ_0058514 in BC patients without distant metastasis and early BC patients (stage 0 and stage 1)

**Figure 3:**
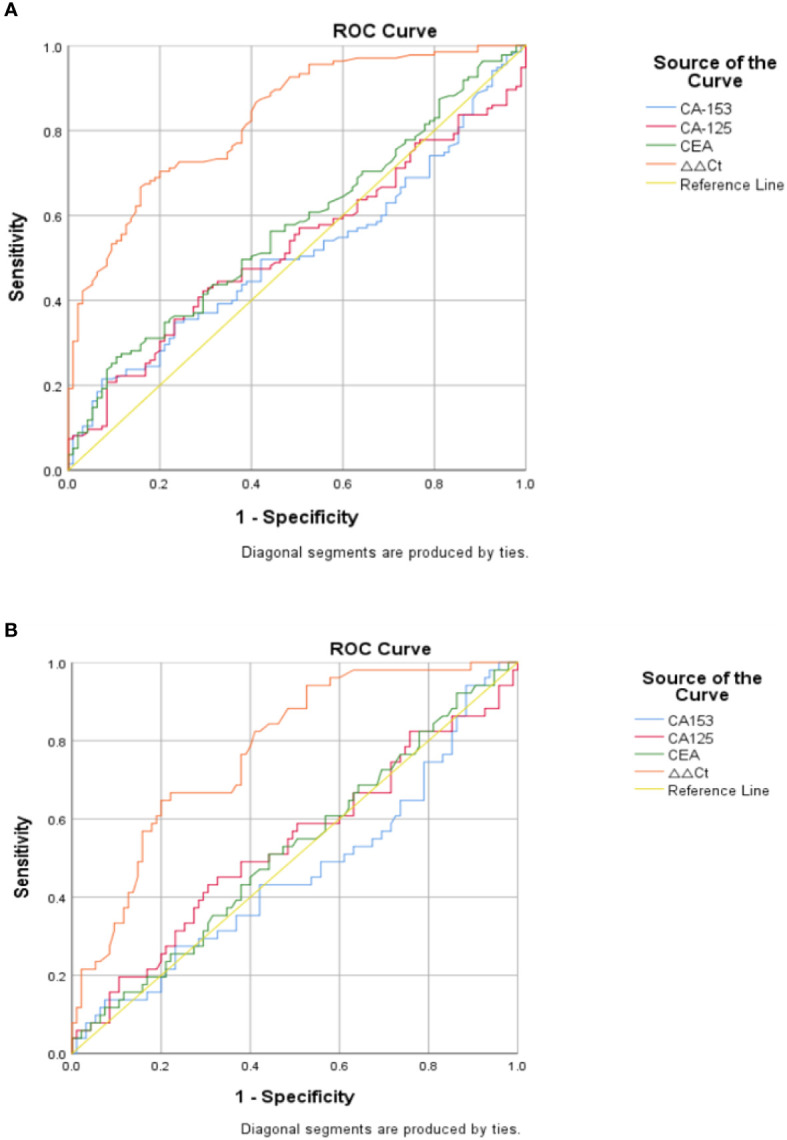
Diagnostic value of hsa_circ_0058514 for BC was evaluated by ROC curve. **(A)** In BC patients without metastasis. **(B)** In early BC patients (stages 0 and 1).

In order to clarify the value of hsa_circ_0058514 expression in the diagnosis of BC patients without metastasis, we performed ROC curve analysis for its plasma expression level, and the results showed that hsa_circ_0058514 expression had better sensitivity (66.7%) and specificity (84.2%), and AUC was 0.828 with a 95% CI of 0.776–0.898. The sensitivity and specificity of CA153 were 21.5% and 92.6%, respectively, and AUC was 0.512 with a 95% CI of 0.437–0.586. CA125 was demonstrated with 42.2% sensitivity and 70.5% specificity, and AUC was 0.527 with a 95% CI of 0.453–0.602. CEA had 26.7% sensitivity and 89.5% specificity, and AUC was 0.573 with a 95% CI of 0.499–0.647 (**Figure 3A**). In early-stage (stage 0 and 1) BC patients, hsa_circ_0058514 showed a higher sensitivity than CA153, CA125, and CEA, which was 64.7% *vs*. 13.7%, 43.1%, and 51.0%. The specificity was also not much lower, 80.0% *vs*. 92.6%, 69.5%, and 55.8%. A higher AUC was also shown in hsa_circ_0058514, which was 0.776 *vs*. 0.463, 0.532, and 0.522; 95% CI was 0.700–0.852, 0.362–0.564, 0.431–0.634, and 0.424–0.621, respectively (**Figure 3B**), which suggested that plasma hsa_circ_0058514 might be used as a good biomarker for the diagnosis of BC.

### Plasma hsa_circ_0058514 can dynamically monitor the effect of neoadjuvant therapy for BC

**Figure 4:**
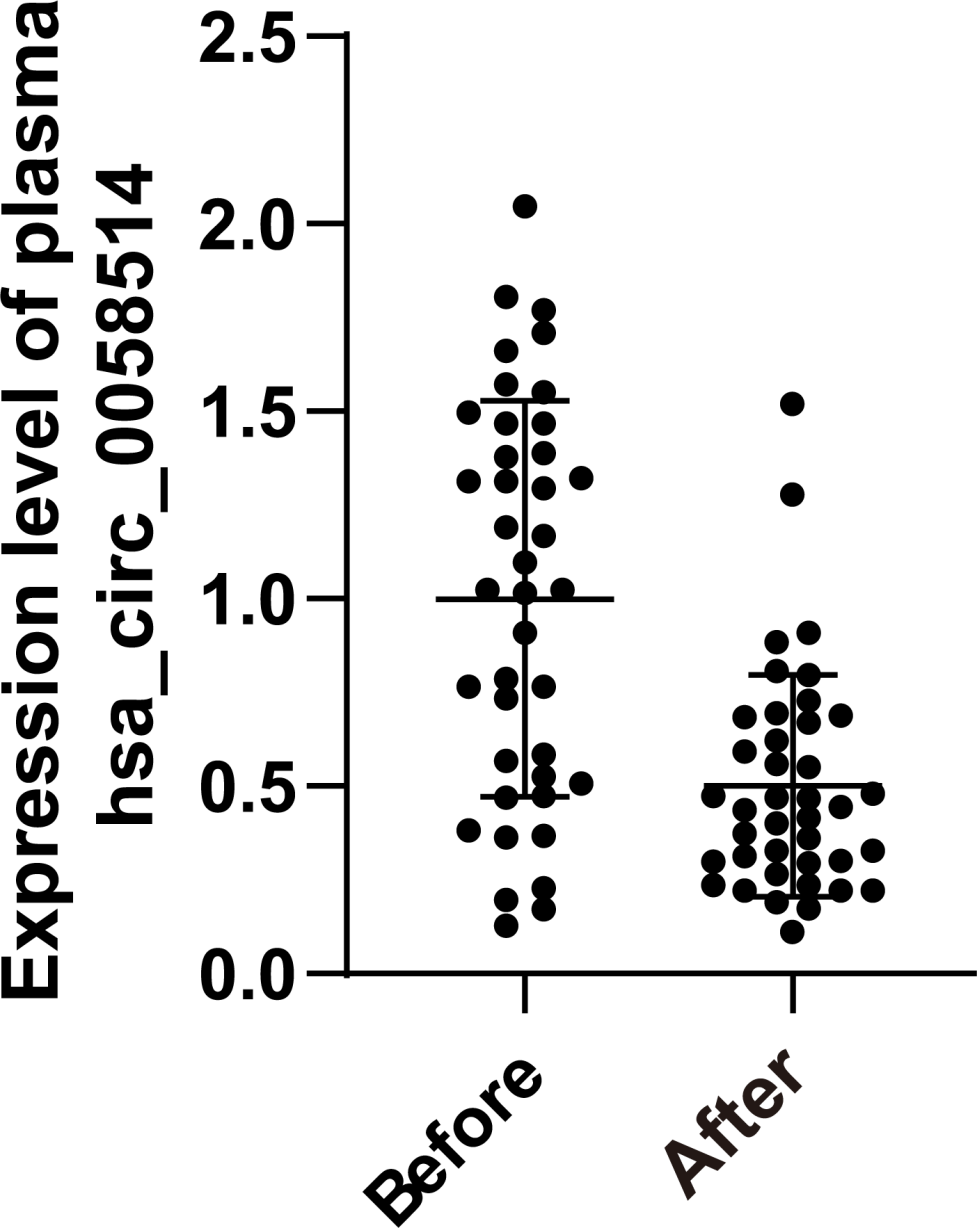
The dynamic monitoring effect of hsa_circ_0058514. The expression level of hsa_circ_0058514 plasma detected in samples of 40 pairs of BC patients before and after neoadjuvant therapy.

Among 40 pairs of BC plasma samples collected before and after neoadjuvant therapy, the expression of hsa_circ_0058514 in plasma significantly decreased (*p* < 0.001) (**Figure 4**). According to the pathological MP grading of neoadjuvant chemotherapy, RESIST grading of imaging target lesions before and after treatment, and histological grading, molecular type, age, tumor stage before treatment, and Ki-67 expression levels were stratified, and the association between the changes of these clinical indicators and the difference of hsa_circ_0058514 before and after treatment was analyzed. The results showed that the decreased expression level of hsa_circ_0058514 was associated with a higher pathological MP grade (*p* = 0.004) and a greater degree of regression of the imaging target lesions (*p* = 0.005), but was not significantly correlated with histological grade, molecular type, age, tumor stage before treatment, and Ki-67 expression (**Table 2**). It is suggested that plasma hsa_circ_0058514 can be used as a predictor of the efficacy of BC treatment.

**Table 2:** The correlation analysis between expression level of hsa_circ_0058514 and clinicopathological features.

Clinicopathological features	*r* value	*p*-value
Subtypes	0.168	0.299
MP grade	0.444	0.004*
RESIST	0.433	0.005*
Histological grade	−0.002	0.993
Age	0.010	0.952
Stage	0.284	0.075
Ki-67 expression level	0.195	0.227

*P < 0.05.

### BC intracellular and EVs encapsulate hsa_circ_0058514 and the detection in plasma

**Figure 5:**
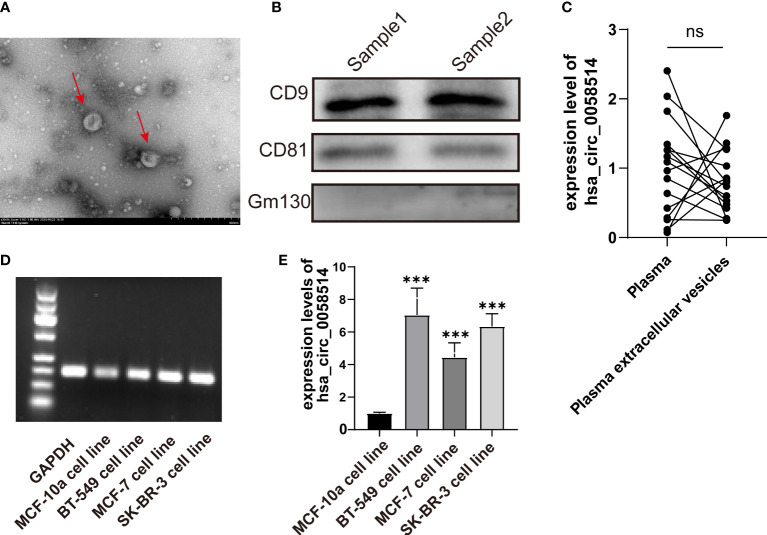
The detection of EVs in BC patients. **(A)** EVs with a diameter of 100–500 nm observed by transmission electron microscopy. **(B)**. Membrane marker proteins CD9 and CD81 detected by Western blotting. **(C)** The detection and **(D)** the quantitative results of hsa_circ_0058514 in EVs of four BC cell lines; the size was 166 bp. **(E)** The expression level of hsa_circ_0058514 in plasma and plasma extracellular vesicle of 16 BC patients. ***P < 0.001; ns, nonsignificant.

*Via* transmission electron microscopy, we observed ectosomes in the serum-free culture medium of BC cells, which were disc-like vesicle-like structures with a diameter of 100–500 nm (**Figure 5A**). The vesicle membrane marker proteins CD9 and CD81 were also proved by Western blotting (**Figure 5B**). The expression of hsa_circ_0058514 from extracellular vesicles was also detected in EVs extracted from the serum-free culture medium of normal breast ductal epithelial MCF-10a cells, and three different BC cell lines including hormone receptor-positive MCF-7 cells, SK-BR-3 cells with Her-2 overexpression, and triple-negative BT-549 cells (**Figure 5C**). Moreover, the expression level of normal mammary duct epithelial cells was significantly lower than that of various BC cells, and the difference was statistically significant (*p* < 0.001) (**Figure 5D**). We further examined the correlation between plasma and plasma extracellular vesicle expression levels in 16 patients with BC, and the qPCR results showed no significant difference between the two groups (**Figure 5E**).

## Discussion

In the present study, it was demonstrated that hsa_circ_0058514 exists in BC patients’ plasma and tumor tissue, and the expression level was associated with lymphatic metastasis, higher tumor stages, recurrence, and non-luminal subtypes. The diagnostic value of plasma hsa_circ_0058514 in early BC patients without distant metastasis was better than CA125, CEA, and CA153, which can also be used to dynamically monitor the effect of neoadjuvant therapy for BC. We also found hsa_circ_0058514 enriched in EVs of plasma and BC tissue.

With the development and widespread application of high-throughput RNA sequencing technology, more evidence suggests that circRNAs might play an important role in the pathogenesis of a variety of diseases, especially the occurrence, development, invasion, metastasis, and drug resistance of cancer (9). Through circRNA chip analysis, it was demonstrated that there were 41 circRNAs with more than two times expression in the plasma of BC patients, of which 19 were upregulated and 22 were downregulated (10). Researchers developed a circular RNA high-throughput workflow to identify unique circular RNAs in breast tumor samples and classify them according to the three BC subtypes (11). An increasing amount of evidence suggests that circRNAs participate in carcinogenesis and the progression of TNBC, which, in turn, can be used as a potential diagnostic and prognostic biomarkers or therapeutic targets for TNBC (12). For example, several upregulated circRNAs like SEPT9, circGNB1, circPGAP3, and hsa_circ_0058514 promote tumor cell proliferation both *in vitro* and *in vivo*, and are associated with larger tumor sizes and shorter survival times for TNBC patients (12, 13). It was also demonstrated that they also contributed to the invasion and metastasis of TNBC cells both *in vitro* and *in vivo*, which might be correlated with advanced TNM stage and poor prognosis of TNBC patients, like circSEPT9, circGNB1, and hsa_circ_0058514 (12, 13). Hsa_circ_0005320 and hsa_circ_0058514 were also associated with decreased cell apoptosis rates of TNBC cells (12, 13). Meanwhile, it was proved that hsa_circ_0058514 significantly promoted tumor angiogenesis *via* CCNEI, associated with positive lymph node metastasis (13). The above-mentioned studies reported the roles of hsa_circ_0058514 in BC, and our study also demonstrated a higher level of expression of hsa_circ_0058514 in BC patients than in healthy subjects.

Notably, hsa_circ_0058514 was detected not only in TNBC, but also in two other subtypes of BC, Her-2 positive and luminal, which revealed its profound clinical application for BC diagnosis. We also compare its diagnostic effect and power with other identified biomarkers, CA153, CA125, and CEA. In addition, we also demonstrate its dynamic monitoring effect for early BC without metastasis. A previous study put forward overexpressed linc-ROR, which might be used as a potential biomarker for BC diagnosis and dynamic monitoring (14). However, this non-coding RNA was also identified in multiple types of cancers, including pancreatic cancer, hepatocellular cancer, bladder cancer, and nasopharyngeal carcinoma (14). Distinct from the above cancer types, hsa_circ_0058514 can be detected in cervical cancer (15), lung cancer (16), colorectal cancer (17), and esophageal cancer (18). Upregulated hsa_circ_0058514 is expressed in cervical cancer tissues and promotes RAF1 expression through the activation of miR-370-3p, which further regulates ovarian cancer progression *via* the RAF/MEK/ERK pathway (15). The hsa_circ_0058514 sponge miR-203 promotes EMT and metastasis of non-small cell lung cancer by upregulating the expression of ZNF28 (16). Hsa_circ_0058514 acts as a sponge of miR-4306 to stimulate the progression of esophageal cancer by regulating the expression of MAPRE2 (18). Hence, the combination of the two biomarkers can be useful for the differential diagnosis of various possible cancers from BC.

In recent years, EVs have received extensive attention as a novel structure vital for intercellular communication mechanism (19). Especially in the field of cancer, there is increasing evidence that EVs play an important role in tumor metastasis and dissemination, for example, the establishment of pre-metastatic niche, angiogenesis, and the formation of cancer-associated fibroblast heterogeneity (20). According to the size and origin of EVs, they can be roughly classified as follows: exosomes (50–200 nm), microvesicles (100–1,000 nm), apoptotic bodies (50–4,000 nm), and prostatic corpuscles (40–500 nm) (19). Furthermore, EVs have been reported to carry a variety of molecules, such as nucleic acids, that reflect the phenotype of their parental cells (19, 21). Since EVs circulate stably in almost all body fluids, they also have great potential as tumor biomarkers (20, 22), even as drug delivery administration systems transferring miRNAs or therapeutic agents to target cells (21). A total of 439 circRNAs detected in plasma EVs with significantly different levels between BC patients and healthy subjects indicated the potential clinical application (23). Moreover, the circRNAs showed temporospatial characteristics that were exhibited in a patient-specific and stage-specific manner, which also implied the relatively more prominent advantage of its specificity (23). It was demonstrated in our preliminary results that hsa_circ_0058514 was encapsulated in EVs, which provided us with a potential mechanism that needs to be investigated as an alternative treatment for BC.

Although this study is limited by its small sample size, our results suggest that upregulation of hsa_circ_0058514 plays an important role in breast cancer development and progression. Large-scale prospective studies should be carried out in the future to verify the accuracy and validity of hsa_circ_0058514 as a representative biomarker for BC. We will further follow up on the prognosis of BC patients to observe the prognostic value of hsa_circ_0058514. In addition, we hope to carry out further animal experiments to further elucidate the secretion mode of exovesicle hsa_circ_0058514 *in vivo*.

## Data availability statement

The original contributions presented in the study are included in the article/**Supplementary Material**. Further inquiries can be directed to the corresponding authors.

## Ethics statement

This study was reviewed and approved by This study was approved by the Ethics Committee of The First Hospital of Qinhuangdao (No. 2022A011 and 2022DW004), and written informed consents were obtained from each study subject. The patients/participants provided their written informed consent to participate in this study.

## Author contributions

YJL, YXQ and MH designed the study. JNL, XYP, XHS and LZ performed the experiments. JXC and WHL collated the data. JZ, TL and QL carried out the data analyses. YY and YZ contributed to drafting the manuscript. All authors have read and approved the final submitted manuscript.

## Acknowledgments

The relevant work of transmission electron microscope imaging was completed in the Electron Microscope Experimental Center of Hebei Medical University. We thank Professor Zhou Chenming for his help in sample preparation and image collection.

## Conflict of interest

Author YZ was employed by Hangzhou Xiaoshan Yaoran Medical Cosmetology Clinic Co. Ltd.

The remaining authors declare that the research was conducted in the absence of any commercial or financial relationships that could be construed as a potential conflict of interest.

## Publisher’s note

All claims expressed in this article are solely those of the authors and do not necessarily represent those of their affiliated organizations, or those of the publisher, the editors and the reviewers. Any product that may be evaluated in this article, or claim that may be made by its manufacturer, is not guaranteed or endorsed by the publisher.
