# Supplementary material for: The value of hsa_circ_0058514 in plasma extracellular vesicles for breast cancer
Source: Front Oncol. 2022 Nov 1;12:995196. doi: 10.3389/fonc.2022.995196 (PMC9663982; doi:10.3389/fonc.2022.995196)
Supplement: Supplementary file 1 [file Table_1.docx]

**Table S1** Sequences of the primers

|  | Forward | Reverse |
| --- | --- | --- |
| Hsa_circ_0058514 | 5ʹ-CCAGTTGTAGGTCGTTCTCAAG-3ʹ | 5ʹ-GGATTTAATCCTCGCCTGCATG-3ʹ |
| GAPDH | 5ʹ-TCGACAGTCAGCCGCATCTTCTTT-3ʹ | 5ʹ-ACCAAATCCGTTGACTCCGACCTT-3ʹ |

Abbreviation: GAPDH: Glyceraldehyde 3-phosphate dehydrogenase
